# Preventive efficacy of 38% silver diamine fluoride and CPP-ACP fluoride varnish on molars affected by molar incisor hypomineralization in children: A randomized controlled trial

**DOI:** 10.12688/f1000research.136653.3

**Published:** 2024-05-07

**Authors:** Zuhair Al-Nerabieah, Muaaz AlKhouli, Mayssoon Dashash

**Affiliations:** 1 Pediatric Dentistry, Damascus University, Damascus, Damascus Governorate, Syria

**Keywords:** Molar-Incisor Hypomineralization, Silver Diamine Fluoride, MI Varnish, Caries prevention, Enamel breakdown, Sensitivity, Children

## Abstract

**Background:**

This randomized controlled trial aimed to compare the efficacy of silver diamine fluoride (SDF) and casein phosphopeptide-amorphous calcium phosphate fluoride varnish (CPP-ACPFV) in preventing caries development, enamel breakdown, and sensitivity on molars affected by molar incisor hypomineralization (MIH) in children.

**Methods:**

A total of 100 children aged 6 to 9 years were enrolled in the study with two contralateral permanent molars mildly affected by MIH. Affected molars were randomly and equally assigned to receive either SDF or CPP-ACPFV treatment. The interventions were applied at four different time points (baseline, 3, 6, 9 months), and the incidence of caries, caries progression, enamel breakdown, and sensitivity were assessed.

**Results:**

The findings of this study revealed significant differences in the incidence of caries between the groups treated with SDF and CPP-ACPFV ( *P*-value < 0.05). Similarly, there was a significant difference in caries progression between the two groups ( *P*-value < 0.05). However, no significant differences were observed in enamel breakdown scores between the treatment groups, as the majority of teeth in both groups exhibited a score of 0. Furthermore, there were no significant differences in sensitivity between the treatment groups throughout the study period.

**Conclusions:**

In conclusion, the results of this study provide evidence that molars treated with SDF demonstrated a lower incidence of caries and a higher rate of caries arrest compared to those treated with CPP-ACPFV. Both interventions showed promise in preventing enamel breakdown and improving sensitivity. These findings highlight the potential of SDF and CPP-ACPFV as effective treatments for caries prevention and management, emphasizing the importance of early intervention and appropriate dental care strategies in maintaining oral health.

**Trial registration:**

ISRCTN54243749 (13/01/2022).

## Introduction

Molar incisor hypomineralization (MIH) is a common dental condition that affects the quality of life of children. ^1^ It is characterized by enamel hypomineralization and discoloration of one or more permanent first molars and incisors, resulting in increased caries and post-eruptive enamel breakdown. ^2^ This can cause a range of oral health problems, including pain and sensitivity, which can affect a child's ability to eat, sleep, and participate in daily activities. ^3^

MIH has become an increasingly recognized problem for researchers and a challenging issue for pediatric dentists. With prevalence rates ranging from 2% to 14% across different populations and diagnostic criteria, MIH poses significant challenges in the field of pediatric dentistry (PD). ^4^ This condition is believed to have a multifactorial etiology, with potential contributing factors including prenatal and perinatal influences, systemic diseases, and environmental exposures. ^5^

Recent research has shed light on the high prevalence of MIH in specific populations. A noteworthy study conducted in Syria revealed a staggering prevalence rate of 39.9% among Syrian children. ^6^ This finding underscores the urgency of addressing MIH and its impact on oral health in affected individuals. Understanding the prevalence of MIH in different populations provides valuable insights into the extent of the problem and emphasizes the need for effective preventive and management strategies. ^7^

Despite the growing recognition of MIH as a significant public health issue, there is limited research available on effective treatments for this condition. ^8^ The impact of MIH on children's quality of life highlights the need for effective treatments that can prevent or mitigate the effects of this condition. ^9^

Although routine oral care practices and fluoride are effective in preventing caries, these methods may not be sufficient in managing MIH. ^8^ MIH-affected teeth have altered enamel structures and are more porous, making them more prone to caries and enamel breakdown than non-affected teeth. The enamel's increased porosity allows bacteria to penetrate and initiate caries formation, leading to early cavitation and further enamel breakdown. ^10^

The consequences of untreated MIH can range from impaired oral function and increased risk of caries, to decreased self-esteem and social isolation. ^11^ As a result, it is important to identify treatments that can effectively prevent or manage MIH and improve the oral health and quality of life of affected children. ^12^

The challenges posed by MIH have led to the development of various preventive and therapeutic measures, such as the use of casein phosphopeptide-amorphous calcium phosphate (CPP-ACP) and silver diamine fluoride (SDF). ^13^ MI Varnish™ is a product that contains 5% NaF and 2% CPP-ACP, which have been shown to enhance enamel remineralization and reduce sensitivity in MIH-affected teeth. ^14^

On the other hand, SDF is a minimally invasive and cost-effective approach for arresting and preventing carious lesions. ^15^ SDF works by inducing the formation of a silver-protein complex and a calcium fluoride-like layer, which can kill cariogenic bacteria and promote remineralization. ^16^ SDF is effective in reducing caries incidence and arresting cavitated lesions in primary and permanent teeth. ^17^^,^^18^ Nevertheless, the efficacy of SDF for preventing caries in MIH-affected molars remains unclear, and there is a lack of studies investigating the use of SDF as a preventive measure for MIH-affected molars.

In this context, the effectiveness of SDF and CPP-ACP in preventing or managing the outcomes of MIH has become an important area of investigation. ^19^ While both SDF and CPP-ACP have been shown to have potential benefits in managing this condition, it is important to understand the relative efficacy of these treatments in a well-controlled, randomized clinical trial. The results of such a trial can inform the development of evidence-based guidelines for the management of MIH in children, and help to ensure that children receive the most effective, and appropriate oral health care.

Therefore, this study aimed to compare the effectiveness of SDF and casein phosphopeptide-amorphous calcium phosphate fluoride varnish (CPP-ACPFV) in preventing the development and progression of caries and post-eruptive enamel breakdown in children's molars affected by MIH.

## Methods

### Ethical considerations

Ethical approval was obtained from the Research Ethics Committee at Damascus University ‘2984’ dated 19/09/2021. Written informed consent was obtained from the parents or legal guardians of all participants before enrolment in the study. The trial was registered on 13/01/2022 at ISRCTN with the following number ( ISRCTN54243749). Furthermore, the study was designed and reported according to the Consolidated Standards of Reporting Trials (CONSORT) statement criteria to ensure rigorous and transparent reporting of the study methodology and results. ^20^

### Study design and setting

This study was a randomized controlled clinical trial, double-blinded with a split-mouth design, conducted in a paediatric dental department at Damascus University, Syria. This study was performed at the period from Jan 2022 to Feb 2023. The first participant was enrolled on Jan 25, 2021, and the last one was enrolled on Feb 21, 2021.

### Sample size determination

The sample size was calculated using G*Power software version 3.1.9.4. Based on the results of a previous study, ^21^ the expected mean difference in the International Caries Detection and Assessment System (ICDAS-II) scores between the two groups was estimated to be 2.2 with a standard deviation of 2.0. The power analysis was conducted to detect significant differences in new caries development, which was the primary outcome measure assessed over a one-year period. Our study was designed as a superiority trial, aiming to determine whether one preventive treatment (SDF or CPP-ACPFV) was superior to the other in preventing new caries development in children with mild MIH.

The power of the study was set at 90%, with an alpha level of 0.05, and a 20% allowance for dropouts. The final sample size was determined to be 100 children (200 permanent molars) between the ages of 6 and 9 years.

Additionally, power analyses were conducted for secondary outcomes, including enamel breakdown and sensitivity assessments. With a sample size of 100 children, the study achieved a power of 0.80 to detect a difference of 1.5 in enamel breakdown scores between the two groups, assuming a standard deviation of 1.2 and a significance level of 0.05. Similarly, for sensitivity assessments, the study achieved a power of 0.85 to detect a difference of 0.8 in sensitivity scores between the groups, assuming a standard deviation of 0.6 and a significance level of 0.05. These power analyses confirm the study's ability to detect clinically meaningful differences in secondary outcomes.

### Inclusion and exclusion criteria

The study's inclusion criteria were carefully defined to ensure a homogeneous sample of participants. Only children between the ages of 6 and 9 years, with two contralateral permanent molars mildly affected by MIH and without any prior treatment on the affected molars, were included. The molars were also required to be fully erupted, with no overlapping gingiva. Additionally, children were required to express their willingness to participate in the study.

Exclusion criteria comprised children with systemic diseases, a history of orthodontic treatment or extractions, silver sensitivity, and allergy to fluoride or milk proteins. Children with other enamel developmental disorders, such as fluorosis and amelogenesis imperfecta, were also excluded from the study. By implementing these criteria, the study aimed to establish a clear and consistent cohort of participants for analysis.

### Randomization and blinding methods

In this split-mouth study, randomization was conducted using two opaque envelopes of different colours to enhance clarity and organization. The first envelope, coloured black, contained two cards for the type of material (SDF/CPP-ACPFV), while the second envelope, coloured red, contained two cards for the side of the mouth (left/right).

To determine the order of application and the tooth to be treated, each eligible patient sequentially drew one card from the black envelope and one card from the red envelope. The first draw determined the type of material to be applied first, while the second draw determined the tooth's side to be treated with the chosen material. This robust randomization process ensured the random allocation of materials to the teeth and minimized the potential for selection bias.

To ensure consistency, the same tooth received the same treatment throughout the study. To minimize any potential bias, both the children and the outcome assessors were blind to the treatment allocation. The children were instructed not to reveal their treatment to the assessors, and the assessors were instructed not to inquire about it. Both SDF and CPP-ACPFV were applied at baseline, and after 3, 6, and 9 months.

### Materials and procedure

The composition of the commercial products, along with their respective lot numbers, is outlined in Table 1 for reference.

**Table 1.** Details of materials used in the study.

Material	Commercial product	Manufacture	Active ingredients	Excipient ingredients	Lot number	Source
38% SDF	Advantage arrest	Elevate Oral Care, USA	25% silver ions and 5% fluoride ions dissolved in an 8% ammonia solution	Water, FD&C Blue No 1	17042	Dental supplier, USA
CPP-ACPFV	Mi Varnish	GC Corporation, Itabashi-Ku, Tokyo, Japan	5% Sodium fluoride, 2% Casein phosphopeptide-amorphous calcium phosphate (CPP-ACP)	Polyvinyl acetate (synthetic resin), Ethanol, Hydrogenated rosin, 1–5% Silicon dioxide, Flavor	191203A	Dental supplier, UAE

An experienced investigator (ZN) was responsible for the examination of children visiting the PD department for MIH, utilizing the European Association of Paediatric Dentistry (EAPD) criteria. ^13^ Parents of eligible children were presented with an informed consent form and an informational brochure regarding the study, with the understanding that participants had the right to withdraw at any time. Only children with two contralateral molars displaying mild MIH lesions were enrolled in the study, characterized by demarcated enamel opacities without caries and enamel breakdown, and had only induced sensitivity to external stimuli.

At the initial visit, both children and parents were provided with dietary advice and oral hygiene instructions. Additionally, participants were given toothbrushes and toothpaste containing 1000-ppm fluoride. In the subsequent visit, sensitivity testing was performed on both affected molars using an air syringe. The air syringe was placed close (1 cm) to the occlusal surface of the affected tooth for one second, and children's response to this stimulus was evaluated using the Schiff Cold Air Sensitivity Scale (SCASS). ^22^ The process was then repeated for the other affected tooth. Furthermore, the Decayed, Missing due to caries, and Filled Teeth (DMFT)/dmft index developed by the World Health Organization was also calculated after documenting the decayed, missing, and filled teeth for both permanent and primary teeth.

SDF (Advantage Arrest, Elevate Oral Care, USA) and CPP-ACPFV (Mi Varnish, GC, Japan) were then applied in the same visit, with the order and side of the application being determined at random by the child. The application procedure for both materials was standardized. Initially, the teeth were cleaned using gauze, and a dry field was maintained using cotton rolls. Subsequently, a single drop of either SDF or CPP-ACPFV was placed in a disposable plastic dish. The material of choice was then applied to the affected tooth using a microbrush applicator (MRG400, Henry Schein, USA) for a duration of one minute. Finally, a layer of petroleum gel (VASELINE, Unilever, USA) was evenly spread over the entire tooth to complete the application process. In addition, parents were provided with specific instructions that participants should avoid the consumption of food or hot beverages for a period of one hour following the application of the preventive materials. This precaution was implemented to ensure the optimal retention and effectiveness of both SDF and CPP-ACPFV.

Sensitivity testing and application of the study materials were conducted again during follow-up visits after 3, 6, 9, and 12 months.

### Assessment of outcome measures

Two pediatric dentist experts were recruited to perform the outcome measures assessment. Prior to the initiation of this study, both examiners underwent comprehensive training and calibration sessions on the International Caries Detection and Assessment System (ICDAS II) criteria and the use of Reveal fluorescence dental loupes. The training regimen encompassed theoretical instruction on ICDAS II and fluorescence in the detection of carious lesions, as well as practical, hands-on training sessions using both carious and sound extracted primary molars. This rigorous training protocol ensured that both examiners were proficient and consistent in their assessment methodologies, thereby enhancing the reliability and validity of the study outcomes.

The primary outcome measures for this study included assessing caries development and caries activity. Caries development was evaluated using the International Caries Detection and Assessment System (ICDAS) criteria and which categorizes tooth surfaces as either sound or carious. The ICDAS outcome was dichotomized into two categories: sound surfaces and carious surfaces. A score of 0 indicated a sound surface without any evidence of caries, while a score of 1 indicated the presence of any detectable signs of caries, regardless of severity. Additionally, caries activity was assessed using Reveal fluorescence dental loupes (Design for vision, USA) and scored as either active or arrested. These scores were recorded at 3, 6, 9, and 12 months from the baseline.

Another primary outcome measure was the assessment of Post eruptive Enamel Breakdown (PEB), which was scored according to the criteria proposed by Ghanim *et al.* (2017). ^23^ PEB scores were recorded at the same time points mentioned previously, and each molar was given a score of 0 for no visible enamel breakdown, 1 for less than one-third of the tooth affected, 2 for at least one-third but less than two-thirds of the tooth affected, and 3 for at least two-thirds of the tooth affected.

In this study, the secondary outcome measure was changes in sensitivity, which were assessed using the SCASS. This scale is a validated tool used to evaluate tooth sensitivity in response to cold stimuli. ^22^ The dental professional responsible for conducting the cold air sensitivity test underwent comprehensive training to ensure proficiency and consistency in test administration. This training encompassed detailed instructions on the correct positioning of the dental air syringe, the duration and intensity of the air stream, and the standardized distance from which the air was delivered to the tooth’s occlusal surface. Moreover, the training emphasized the importance of clear communication with the children to ensure their understanding of the procedure and to encourage accurate reporting of any sensations experienced during the test.

Additionally, the dental professional received guidance on recognizing and documenting the various response categories outlined in the SCASS scale, thereby ensuring uniform interpretation and scoring of sensitivity levels. Overall, the specific training regimen aimed to equip the dental professional with the necessary skills and knowledge to conduct the sensitivity test reliably and consistently across all study participants.

Before the testing, children were informed about the procedure and what to expect. They were instructed to report any discomfort or sensation experienced during the test.

The child was introduced to the sensitivity test using a standardized script in Arabic, which was translated into English for clarity. The script used for the introduction was as follows:

Orginal Arabic script:

“مرحبا، اليوم سنقوم بإجراء اختبار للحساسية على أسنانك. سأقوم بتوجيه هواء بارد إلى أسنانك لمدة قصيرة وسأطلب منك أن تخبرني عن أي شعور تشعر به. إذا شعرت بأي ألم أوانزعاج،يرجى إخباري بذلك فورًا. هل لديك أي أسئلة قبل أن نبدأ؟”

English Translation:

“Hello, today we will conduct a sensitivity test on your teeth. I will direct some cold air to your teeth for a short period, and I will ask you to tell me about any sensation you feel. If you experience any pain or discomfort, please let me know immediately. Do you have any questions before we begin?”

This script was designed to standardize the introduction of the sensitivity test to ensure consistency in the procedure across all participants. Additionally, the dental staff delivering the test received training to ensure that the script was delivered in a clear and consistent manner to every child involved in the study.

During the assessment, a standard dental air syringe was used to deliver a stream of air for 1 second at a distance of 1 cm and perpendicular to the occlusal surface of the tooth. The subject's response to this stimulus was observed and evaluated using the SCASS Scale. The scale consists of four response categories: 0 represents no response to the stimulus, 1 indicates no response to the stimulus but the subject considers it painful, 2 signifies a response to the stimulus with the subject moving away from it, and 3 indicates a response to the stimulus with the subject moving away and requesting immediate discontinuation. The subject's response was recorded and scored accordingly, in which a higher score indicates a greater sensitivity to cold stimuli.

Sensitivity scores were recorded at 3, 6, 9, and 12 months from the baseline, allowing for the evaluation of changes in sensitivity over time. Additionally, the dental professional performing the test at follow-up was blinded to the results from the earlier period to prevent any bias in the assessment of sensitivity changes over time.

### Data analysis and examiner reliability

Data were analyzed using IBM SPSS version 23 (IBM Corp., Armonk, USA). Descriptive data including the distribution of affected molars were demonstrated. *Chi-squared* test was used to analyze the difference between the two studied groups regarding the development of caries in the MIH molars and the activity of the caries lesions at each time point (3 months, 6 months, 9 months, and 12 months). In addition, *repeated measures ANOVA* test was performed to detect if there was a difference between the different time points in each group separately. However, the *Bonferroni* test was the post hoc test used to determine where the difference was. The P-value of 0.05 was considered the level of significance. The mean rank of the scores of enamel breakdown and SCASS were calculated to conduct the *Mann-Whitney U* test to reveal the difference between the two groups at each time point. Similarly, *repeated measures ANOVA* test was performed to detect if there was a difference between the different time points in each group. *Wilcoxon signed Rank* test was used as a post hoc test to detect where the difference was. *Friedman* test was used to reveal the difference between the four-time points regarding the mean rank of enamel breakdown scores in both groups. The intra-examiner reliability was assessed through the re-examination of 10% of the participants one week after the initial examination and was found to be high ( *Kappa* coefficient = 0.86). The inter-examiner reliability was assessed by independent examination of a sample of participants and was found to be high ( *Kappa* coefficient = 0.87).

## Results

A total of 100 children were enrolled in this trial (42 girls and 58 boys) with a mean age of 7.6 ± 1.4. Each child had two contralateral permanent 1 ^st^ molars affected with mild MIH. ^24^ The distribution of the molars involved in the study is summarized in Table 2.

**Table 2.** The distribution of the molars involved in the study.

	Boys	Girls	Total
**Maxillary molars**	26	44	70 (35%)
**Mandibular molar**	90	40	130 (65%)
**Total**	116 (58%)	84 (42%)	200 (100%)

The mean and standard deviation of dmft and DMFT indices were 4.37 ± 1.40 and 0.30 ± 0.63, respectively. The proportion of caries-free children was 11.3%. Table 3 shows the number of molars that were affected by caries in each group and for the four studied time points (after 3, 6, 9, and 12 months). The *chi-squared* test showed that carious molars were less in the SDF group than in the MI group with a statistically significant difference ( *P*-value < 0.05) in each follow-up schedule.

**Table 3.** The difference between groups and within groups regarding caries development.

Intervention		3 months	6 months	9 months	12 months	Sum of squares	F-value	*P*-value
**SDF**	Caries (number of teeth)	11	15	21	23	91	0.42	0.183
	No caries (number of teeth)	89	85	79	77			
**CPP-ACPFV**	Caries (number of teeth)	33	39	44	49	140.74	0.32	0.111
	No caries (number of teeth)	67	61	56	51			
**Chi-squared**		2.621	4.489	7.562	9.141			
***P*-value**		0.002 *	0.034 *	0.023 *	0.001 *			

SDF: Silver diamine fluoride.

* Significant difference.

On the other hand, *repeated measures ANOVA* showed that there was no statistically significant difference in the development of caries between the four-time points in neither the SDF group nor the MI group ( *P*-value > 0.05).

**Figure 1.**
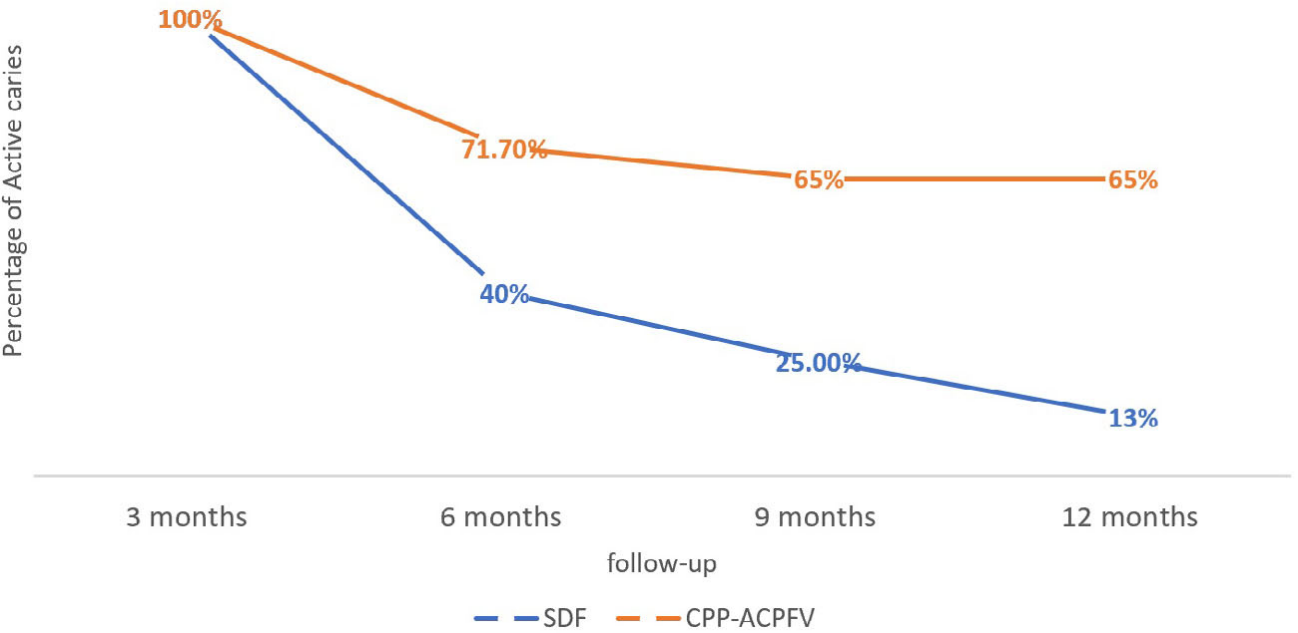
The difference between the percentage of active caries in both groups. (SDF: Silver diamine fluoride, CPP-ACPFV: Casein phosphopeptide-amorphous calcium phosphate fluoride varnish).

Moreover, *the chi-squared* test showed that the percentages of arrested caries were higher in the SDF group than in the MI group with a statistically significant difference in months 6, 9, and 12 ( *P*-value < 0.05). This means that our findings revealed that SDF is better than CPP-ACPFV in arresting the developed caries lesions, especially after 6 months of application ( Figure 1). Additionally, *repeated measures ANOVA* test detected that there was a statistically significant difference between the four-time points in arresting caries in both studied groups ( Table 3).

*Bonferroni post hoc* test indicated that the difference was statistically significant between the 3 months follow-up and the other time points only.

Regarding the enamel breakdown detected in each group, the *Mann-Whitney U* test showed that there was no statistically significant difference between the SDF and MI groups at each time point. This means that both SDF and CPP-ACPFV were effective in the prevention of enamel breakdown of MIH molars ( Table 4).

**Table 4.** The difference between groups and within groups regarding arresting the carious lesions.

Intervention		3 months	6 months	9 months	12 months	Sum of squares	F-value	*P*-value
**SDF**	Active caries (number of teeth)	11 (100%)	6 (40%)	5 (25%)	3 (13%)	222	1.22	0.002 *
	Arrested caries (number of teeth)	0 (0%)	9 (60%)	15 (75%)	20 (87%)			
**CPP-ACPFV**	Active caries (number of teeth)	33 (100%)	28 (71.7%)	29 (65%)	32 (65%)	173.75	1.45	0.001 *
	Arrested caries (number of teeth)	0 (0%)	11 (28.3%)	15 (34%)	17(34.7%)			
**Chi-squared**		0.193	1.431	4.507	9.252			
***P*-value**		0.660	0.021 *	0.034 *	0.002 *			

SDF: Silver Diamine fluoride.

* Significant difference.

*Friedman* test indicated that there was no statistically significant difference between the four-time points regarding the mean rank of enamel breakdown scores in neither the SDF group nor in the MI group ( *P*-value= 0.513, 0.221 respectively) ( Table 5).

**Table 5.** Mann-Whitney U test to study the difference between the groups regarding enamel breakdown.

Enamel breakdown	3 months		6 months		9 months		12 months	
	SDF	CPP-ACPFV	SDF	CPP-ACPFV	SDF	CPP-ACPFV	SDF	CPP-ACPFV
**0 (number of teeth)**	99	100	98	99	95	97	92	91
**1 (number of teeth)**	1	0	0	1	2	1	2	4
**2 (number of teeth)**	0	0	2	0	2	0	4	3
**3 (number of teeth)**	0	0	0	0	1	2	2	2
**Mean rank**	11	12	10	12	12	13	14	15
***P*-value**	0.732		0.453		0.231		0.322	

SDF: Silver diamine fluoride.

As shown in Table 6, *the Mann-Whitney U* test similarly showed that there was no statistically significant difference between the SDF group and the MI group regarding the Schiff Cold Air Sensitivity Scale at each time point. However, *the Friedman* test indicated that the difference between the five-time points (baseline, 3 months, 6 months, 9 months, and 12 months) was statistically significant in both SDF and MI groups.

**Table 6.** Mann-Whitney U test to study the difference between the groups regarding SCASS.

SCASS	Baseline		3 months		6 months		9 months		12 months	
	SDF	CPP-ACPFV	SDF	CPP-ACPFV	SDF	CPP-ACPFV	SDF	CPP-ACPFV	SDF	CPP-ACPFV
**0 (number of teeth)**	55	56	69	67	80	77	83	83	84	83
**1 (number of teeth)**	33	32	25	26	18	19	17	15	16	15
**2 (number of teeth)**	7	6	4	5	2	3	0	2	0	2
**3 (number of teeth)**	5	6	2	3	0	1	0	0	0	0
**Mean Rank**	16	15	13	15	12	13	11	11	11	10
***P*-value**	0.454		0.421		0.309		0.288		0.122	

SCASS: Schiff cold air sensitivity scale; SDF: Silver diamine fluoride.

**Figure 2.**
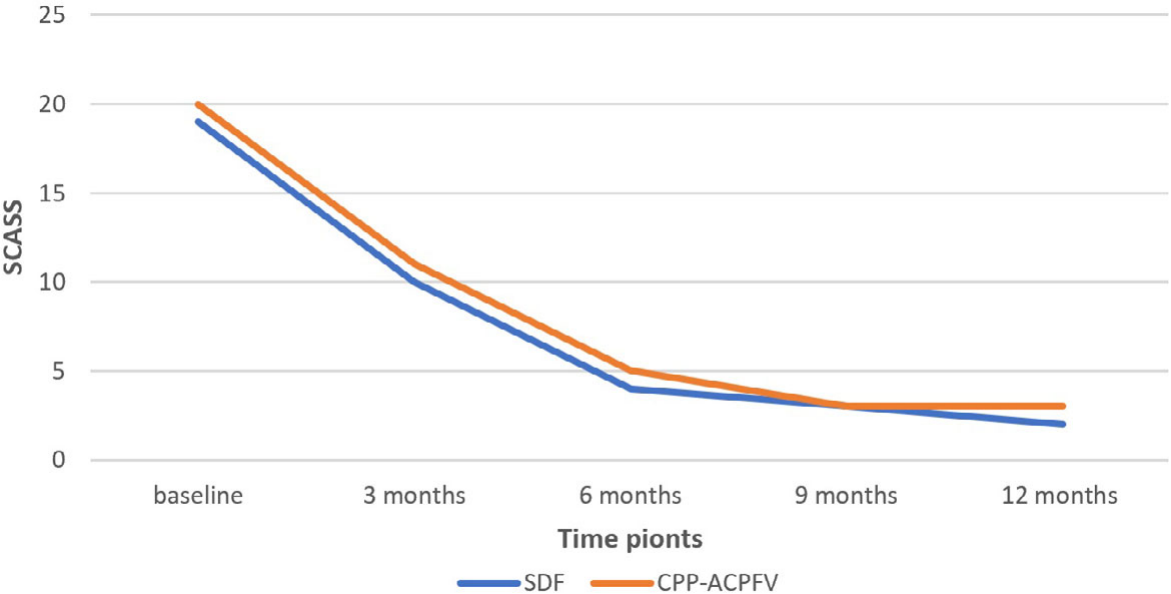
The change in the mean rank of Schiff cold air sensitivity scale SCASS between the groups and between the time points. (SDF: Silver diamine fluoride, CPP-ACPFV: Casein phosphopeptide-amorphous calcium phosphate fluoride varnish).

*Wilcoxon Signed Rank* test revealed that in both SDF and MI groups, there was a statistically significant difference between the baseline and 3 months (P-value = 0.002, 0.001, respectively), between the baseline and 6 months of application (P-value = 0.001, 0.021, respectively) and between the 3 months and 6 months of application (P-value = 0.011, 0.001, respectively). However, there was no statistically significant difference between 9 months and 12 months neither in the SDF group nor in the MI group. Figure 2 shows the change in the mean rank of SCASS between the groups and between the time points.

## Discussion

This study aimed to evaluate and compare the preventive efficacy of SDF and CPP-ACPFV in molars affected by MIH among children aged 6–9 years. The results of this study indicate that molars treated with SDF had a significantly lower incidence of caries and a higher rate of caries arrest compared to those treated with CPP-ACPFV. Both SDF and CPP-ACPFV demonstrated the ability to prevent enamel breakdown and improve sensitivity, with no statistically significant difference between the two treatments.

This age group was selected based on several considerations related to the nature of MIH and the developmental stage of affected teeth.

MIH commonly manifests during the eruption of the first permanent molars, which typically occurs around the age of 6 years. ^25^ By including children within the age range of 6–9 years, the study focused on a critical period when MIH-affected molars are fully erupted and accessible for examination and treatment.

Furthermore, children within this age range are more likely to comply with study protocols and express their willingness to participate actively. They have a better ability to understand and cooperate during dental examinations and treatments, enhancing the feasibility of the study. Additionally, by targeting this age group, the study aimed to address the preventive needs of children at a crucial developmental stage when early intervention can have a significant impact on long-term oral health outcomes. ^26^^,^^27^

The utilization of the European Academy of Pediatric Dentistry (EAPD) criteria for the diagnosis and classification of MIH played a significant role in this study. The EAPD criteria have been widely accepted and recommended as the gold standard for the diagnosis and classification of MIH, providing a standardized approach to ensure consistency and comparability among studies and clinical practice. ^13^

In addition, the implementation of the EAPD criteria for inclusion ensured the homogeneity of the sample and the accurate identification of participants with MIH. By applying these criteria, the researchers could confidently select children with mild MIH-affected molars, minimizing potential confounding factors and enhancing the validity of the study results.

In the context of this study, the application of petroleum gel following treatment serves multiple purposes. Primarily, it acts as a barrier to prevent the overlapping effect of the treatment material from one side of the mouth to the other. This is particularly relevant in split-mouth designs, where different materials are applied to different sides of the mouth. By creating a physical barrier, Vaseline helps to ensure that there is no inadvertent transfer of the treatment material, thereby maintaining the integrity of the study design and minimizing the risk of cross-contamination between treatment groups. Additionally, Vaseline aids in ensuring the even distribution and retention of the applied material on the tooth surface, thereby maximizing its therapeutic effects.

While the use of petroleum gel deviates from standard clinical practice, its inclusion in our study protocol was necessary to ensure the validity of our findings. However, it is essential to acknowledge that this deviation may introduce a confounding factor that could potentially influence the study outcomes. Therefore, future research should consider the implications of using Vaseline as part of the treatment protocol and explore alternative methods to mitigate any associated biases.

In the context of this study, the ICDAS-II criteria were employed to detect and assess caries incidence in the molars affected by MIH. ICDAS-II is considered a widely recognized and standardized method for detecting and assessing caries. ^28^ It provides a comprehensive and detailed classification system that allows for the consistent evaluation and diagnosis of caries lesions based on their visual appearance.

In addition, the caries activity outcome measure was assessed using Reveal fluorescence dental loupes, and the results showed that SDF had a higher percentage of arresting caries compared to CPP-ACPFV, and this difference was found to be statistically significant.

The use of Reveal fluorescence dental loupes for evaluating caries activity in this study provided an additional objective measure to assess the effectiveness of the preventive treatments. ^29^ These dental loupes utilize fluorescence technology to detect the presence of caries activity based on the fluorescence emission of bacterial metabolites in the oral cavity. ^30^

The inclusion of Reveal fluorescence dental loupes offered several advantages in this study. Firstly, it is a non-invasive and painless procedure, making it well-suited for use in pediatric patients. Secondly, the immediate results provided a real-time assessment of caries activity, enabling prompt decision-making regarding treatment interventions. Additionally, the qualitative nature of the fluorescence measurements contributed to more objective and standardized evaluations, reducing subjectivity in the assessment process. ^29^^,^^30^

Furthermore, a recent study by Steier and colleagues, focusing on fluorescent enhanced theragnosis through Reveal Vision glasses for the diagnosis of caries progress. Their study revealed a substantial kappa score of 0.706, indicating significant reproducibility for the given diagnostic technique. ^31^

The superior performance of SDF in preventing and arresting caries can be attributed to its unique mechanism of action. SDF works by inducing the formation of a silver-protein complex and a calcium fluoride-like layer, which effectively kills cariogenic bacteria and promotes remineralization. ^16^ This antimicrobial and remineralization effect of SDF is particularly beneficial in managing caries in MIH-affected molars, where the enamel is more susceptible to demineralization and cavitation. ^32^

The significant difference observed between SDF and CPP-ACPFV suggests that SDF may be a more effective preventive treatment option for caries in MIH-affected molars. These findings align with previous studies that have demonstrated the superiority of SDF in caries prevention and arrest, particularly in primary and permanent teeth. ^33^^,^^34^

Despite the growing interest in the effectiveness of CPP-ACP in preventing caries development and progression, there is still ongoing research in this area. In line with previous studies, our research findings support the notion that CPP-ACP demonstrates efficacy in preventing caries. A recent systematic review and meta-analysis conducted by Sharda *et al.* (2020) investigated the efficacy of CPP-ACP complex and topical fluoride (TF) in managing caries. ^35^ The study revealed that while CPP-ACP showed superiority over TF monotherapy in remineralizing existing lesions and exhibiting a stronger antibacterial effect, it did not demonstrate greater efficacy in preventing caries incidence.

The findings of Sharda *et al.*'s systematic review highlight the limitations of CPP-ACP in terms of preventing the occurrence of new carious lesions. Although CPP-ACP has shown promise in remineralizing existing lesions and exerting antibacterial properties, its impact on preventing caries incidence appears to be limited. ^35^

Furthermore, a recent study showed that a single topical application of the GC Tooth mousse (CPP-ACP) does not affect increasing the salivary pH and salivary flow rate which are great factors in caries prevention. ^36^

On the other hand, both SDF and CPP-ACPFV showed comparable performance in preventing enamel breakdown (PEB). This indicates that both materials were effective in preserving the integrity of the enamel surface and preventing further deterioration of the affected teeth. The lack of significant difference in PEB outcomes between the two groups suggests that both treatments have a similar capacity to stabilize the enamel and prevent its breakdown.

Furthermore, both SDF and CPP-ACPFV demonstrated improvement in sensitivity, with no significant difference observed between the two groups. This indicates that both materials were effective in alleviating the sensitivity commonly associated with MIH-affected molars. The reduction in sensitivity can greatly improve the quality of life for affected children by minimizing discomfort and facilitating normal oral function.

The efficacy of CPP-ACP in preventing enamel breakdown and improving sensitivity has been investigated in several studies, allowing for a comparison of results. In our study, we found that CPP-ACPFV demonstrated effectiveness in preventing enamel breakdown. This finding is consistent with previous research that has reported positive outcomes with the use of CPP-ACPFV as a preventive treatment for enamel hypomineralization.

A notable investigation conducted by Bakkal *et al.* (2017) aimed to assess the efficacy of 10% CPP-ACP cream in enhancing the mineral content of hypomineralized enamel. The outcomes of their study demonstrated a marked improvement in mineralization, as well as enhancements in enamel morphology and reductions in porosities. ^37^ This highlights the positive impact of CPP-ACP cream on addressing the deficiencies in hypomineralized enamel.

Moreover, Cardoso-Martins *et al.* (2022) conducted a study that further supports these findings. Their investigation focused on evaluating the effects of CPP-ACP tooth mousse on the mineral density and organization of hypomineralized enamel. The results demonstrated significant improvements in both mineral density and the overall structural organization of the affected enamel following treatment with CPP-ACP tooth mousse. ^38^ This study reinforces the potential of CPP-ACP as a valuable therapeutic option for ameliorating the compromised enamel associated with hypomineralization.

Regarding the improvement of sensitivity, our study demonstrated that CPP-ACPFV was effective in alleviating sensitivity associated with MIH. Similarly, a systematic review by Somani *et al.* (2022) analyzed multiple studies and concluded that CPP-ACP products showed promising results in reducing sensitivity in MIH-affected teeth. ^8^ They reported a significant reduction in sensitivity scores following the application of MI Varnish.

Overall, the results of our study, along with the findings of previous research, support the use of CPP-ACPFV as an effective intervention for preventing enamel breakdown and improving sensitivity in individuals with MIH.

Several limitations should be considered when interpreting the findings of this study. Firstly, the study sample was drawn from a specific population of children aged 6 to 9 years with MIH, which may restrict the generalizability of the results to other age groups or individuals with different characteristics. Further research involving diverse populations is necessary to validate the findings across a broader range of individuals affected by MIH.

In addition, the study had a relatively short follow-up period, which may limit the ability to assess the long-term effectiveness and durability of the preventive treatments. Hence, longer follow-up periods would provide valuable insights into the sustainability of the interventions over time.

Furthermore, it’s important to acknowledge that the utilization of a split-mouth design introduced certain limitations that warrant discussion. One notable concern is the potential for treatment administered on one side to inadvertently influence the outcomes on the contralateral side.

We acknowledge this possibility and took several steps to minimize its impact. Firstly, meticulous application techniques were employed to ensure precise placement of the treatment materials on the designated teeth. Additionally, petroleum jelly was applied to the teeth after treatment to prevent material spread and minimize the risk of crossover effects.

While these measures were implemented to mitigate the limitations inherent to split-mouth designs, we recognize that some degree of treatment crossover may still have occurred. Therefore, it is essential to interpret the study findings with caution, considering the potential for treatment crossover when evaluating the outcomes of split-mouth studies.

An additional limitation of this study was the inability to blind the investigator who applied the intervention materials (SDF and CPP-ACPFV) to the participants. Blinding is a methodological approach that aims to reduce bias by withholding information about the assigned treatment from the investigator. However, in this study, blinding was not feasible due to the distinct differences in the colour and texture of the intervention materials.

## Conclusion

In summary, this study aimed to evaluate and compare the preventive efficacy of SDF and CPP-ACPFV in molars affected by MIH among children aged 6 to 9 years. The results demonstrated that molars treated with SDF had a lower incidence of caries and a higher rate of caries arrest compared to those treated with CPP-ACPFV. Both interventions showed promise in preventing enamel breakdown and improving sensitivity, with no significant difference observed between them.

The findings suggest that SDF may be a valuable preventive treatment option for managing caries in MIH-affected molars. Furthermore, the finding of this study suggests that while CPP-ACP may be beneficial for managing sensitivity and enhancing remineralization, additional preventive measures may be necessary to address the prevention of new caries formation.

However, it is important to acknowledge the limitations of this study, including the restricted sample and short follow-up period. Future research with larger and more diverse populations, as well as longer-term assessments, is needed to further validate these findings.

## Data Availability

Mendeley Data: Preventive Efficacy of Silver Diamine Fluoride and MI Varnish on Molars Affected by Molar Incisor Hypomineralization in Children: A Randomized Controlled Trial. https://doi.org/10.17632/xtw9gvhbfp.2. ^24^ This project contains the following underlying data:
• Ethical approval.jpeg • Patient data entry level.xlsx (This file includes Age, gender and teeth postion (U upper jaw – L lower jaw) for each participant) • Protocol.pdf (This file includes the full protocol of this study with analysis plan) • Teeth data entry level.xlsx (This file includes baseline data of participants’ teeth) • Outcome assessment Scores 3m, 6m, 9m and 12m.xlsx (This file includes all raw data and scores of assessment scales used in this study for all the follow up periods) Menedeley Data: CONSORT flow diagram and checklist for ‘Preventive efficacy of silver diamine fluoride and MI Varnish on molars affected by molar incisor hypomineralization in children: A randomized controlled trial’ https://doi.org/10.17632/6yx6my74ys.2. ^20^ Data are available under the terms of the Creative Commons Attribution 4.0 International license (CC-BY 4.0).

## References

[1] Portella PD : Impact of molar incisor hypomineralization on quality of life in children with early mixed dentition: a hierarchical approach. *Int. J. Paediatr. Dent.* 2019;29:496–506. 10.1111/ipd.12482 30758096

[2] Weerheijm KL Jälevik B Alaluusua S : Molar-Incisor Hypomineralisation. *Caries Res.* 2001;35:390–391. 10.1159/000047479 11641576

[3] Weerheijm KL : Molar Incisor Hypomineralisation (MIH). *Eur. J. Paediatr. Dent.* 2003;4:115–120. 14529330

[4] Zhao D Dong B Yu D : The prevalence of molar incisor hypomineralization: evidence from 70 studies. *Int. J. Paediatr. Dent.* 2018;28:170–179. 10.1111/ipd.12323 28732120

[5] Juárez-López MLA Salazar-Treto LV Hernández-Monjaraz B : Etiological Factors of Molar Incisor Hypomineralization: A Systematic Review and Meta-Analysis. *Dent. J.* 2023;11:111. 10.3390/dj11050111 37232762 PMC10217283

[6] Al-Nerabieah Z AlKhouli M Dashash M : Prevalence and Clinical characteristics of Molar-Incisor Hypomineralization in Syrian children: A Cross-sectional Study. *Sci. Rep.* 2023;13:8582. 10.1038/s41598-023-35881-3 37237023 PMC10219925

[7] Bandeira Lopes L Machado V Botelho J : Molar-incisor hypomineralization: an umbrella review. *Acta Odontol. Scand.* 2021;79:359–369. 10.1080/00016357.2020.1863461 33524270

[8] Somani C : An update of treatment modalities in children and adolescents with teeth affected by molar incisor hypomineralisation (MIH): a systematic review. *Eur. Arch. Paediatr. Dent.* 2022;23:39–64. 10.1007/s40368-021-00635-0 34110615 PMC8927013

[9] Gevert MV : How is the quality of the available evidence on molar-incisor hypomineralization treatment? An overview of systematic reviews. *Clin. Oral Investig.* 2022;26:5989–6002. 10.1007/s00784-022-04612-9 35790597

[10] Mahoney EK Farah R : Molar Incisor Hypomineralization: Structure, Composition, and Properties. *Planning and Care for Children and Adolescents with Dental Enamel Defects: Etiology, Research and Contemporary Management.* Berlin Heidelberg: Springer;2015;73–84. 10.1007/978-3-662-44800-7_6

[11] Rodd HD Graham A Tajmehr N : Molar incisor hypomineralisation: current knowledge and practice. *Int. Dent. J.* 2020;71:285–291. 10.1111/idj.12624 34286697 PMC9275314

[12] Jälevik B Sabel N Robertson A : Can molar incisor hypomineralization cause dental fear and anxiety or influence the oral health-related quality of life in children and adolescents?—A systematic review. *Eur. Arch. Paediatr. Dent.* 2022;23:65–78. 10.1007/s40368-021-00631-4 34110616 PMC8927003

[13] Lygidakis NA : Best clinical practice guidance for clinicians dealing with children presenting with molar-incisor-hypomineralisation (MIH): An updated European Academy of Paediatric Dentistry policy document. *Eur. Arch. Paediatr. Dent.* 2022;23:1–19. 10.1007/s40368-022-00693-y 34669177 PMC8926988

[14] Haradwala Z Winnier J : Aesthetic Management of MIH Affected Young Permanent Anterior Teeth: A Review. *Adv. Dent. J.* 2022;4:149–159. 10.21608/adjc.2022.103221.1118

[15] Seifo N Cassie H Radford JR : Silver diamine fluoride for managing carious lesions: an umbrella review. *BMC Oral Health.* 2019;19:1–10. 10.1186/s12903-019-0830-5 31299955 PMC6626340

[16] Zhao IS : Mechanisms of silver diamine fluoride on arresting caries: a literature review. *Int. Dent. J.* 2018;68:67–76. 10.1111/idj.12320 28542863 PMC9378923

[17] Oliveira BH Rajendra A Veitz-Keenan A : The effect of silver diamine fluoride in preventing caries in the primary dentition: A systematic review and meta-analysis. *Caries Res.* 2019;53:24–32. 10.1159/000488686 29874642 PMC6292783

[18] Crystal YO Niederman R : Evidence-Based Dentistry Update on Silver Diamine Fluoride. *Dent. Clin. N. Am.* 2019;63:45–68. 10.1016/j.cden.2018.08.011 30447792 PMC6500430

[19] Enax J : Remineralization Strategies for Teeth with Molar Incisor Hypomineralization (MIH): A Literature Review. *Dent. J.* 2023;11:80. 10.3390/dj11030080 36975577 PMC10047667

[20] Al-Nerabieah Z Alkhouli M Dashash M : Preventive Efficacy of Silver Diamine Fluoride and MI Varnish on Molars Affected by Molar Incisor Hypomineralization in Children: A Randomized Controlled Trial (CONSORT flow diagram and CONSORT checklist). *Mendeley Data.* 2023;V2. 10.17632/6yx6my74ys.2 PMC11109571 38778809

[21] Schraverus MS : Glass ionomer sealants can prevent dental caries but cannot prevent posteruptive breakdown on molars affected by molar incisor hypomineralization: one-year results of a randomized clinical trial. *Caries Res.* 2021;55:301–309. 10.1159/000516266 34107492 PMC8491481

[22] Gernhardt CR : How valid and applicable are current diagnostic criteria and assessment methods for dentin hypersensitivity? An overview. *Clin. Oral Investig.* 2013;17:31–40. 10.1007/s00784-012-0891-1 23224044 PMC3585843

[23] Ghanim A : Molar incisor hypomineralisation (MIH) training manual for clinical field surveys and practice. *Eur. Arch. Paediatr. Dent.* 2017;18:225–242. 10.1007/s40368-017-0293-9 28721667

[24] Al-Nerabieah Z Alkhouli M Dashash M : Preventive Efficacy of Silver Diamine Fluoride and MI Varnish on Molars Affected by Molar Incisor Hypomineralization in Children: A Randomized Controlled Trial. *Mendeley Data.* 2023;V2. 10.17632/xtw9gvhbfp.2 PMC11109571 38778809

[25] Almuallem Z Busuttil-Naudi A : Molar incisor hypomineralisation (MIH)–an overview. *Br. Dent. J.* 2018;225:601–609. 10.1038/sj.bdj.2018.814 30287963

[26] Ballouk MA-H Dashash M : Caries prevalence and dental health of 8–12 year-old children in Damascus city in Syria during the Syrian Crisis; a cross-sectional epidemiological oral health survey. *BMC Oral Health.* 2019;19:1–6. 10.1186/s12903-019-0713-9 30646889 PMC6332908

[27] Ballouk MA-H Dashash M : The gingival health status of 8–12 year-old children in Damascus city in Syria during the Syrian Crisis: a cross-sectional epidemiological oral health survey. *BMC. Res. Notes.* 2018;11:1–5. 30545414 10.1186/s13104-018-3998-x PMC6293583

[28] Foros P Oikonomou E Koletsi D : Detection methods for early caries diagnosis: A systematic review and meta-analysis. *Caries Res.* 2021;55:247–259. 10.1159/000516084 34130279

[29] Steier L Figueiredo JAP de Blatz MB : Fluorescence-Enhanced Theragnosis: A Novel Approach to Visualize, Detect, and Remove Caries. *Compendium.* 2021;42:460–465. 34449243

[30] Steier L : Reveal: Fluorescence Enhanced Theragnosis by Designs for Vision. *Eur. J. Dent.* 2020;14:186–188. 10.1055/s-0040-1705076 32168545 PMC7069761

[31] Steier L Sidhu P Qasim SS : Visualization of initial bacterial colonization on dentin using fluorescence activating headlight for fluorescence enhanced theragnosis. *Photodiagnosis Photodyn. Ther.* 2022;38: 102732. 10.1016/j.pdpdt.2022.102732 35066134

[32] Ballikaya E Ünverdi GE Cehreli ZC : Management of initial carious lesions of hypomineralized molars (MIH) with silver diamine fluoride or silver-modified atraumatic restorative treatment (SMART): 1-year results of a prospective, randomized clinical trial. *Clin. Oral Investig.* 2022;26:2197–2205. 10.1007/s00784-021-04236-5 PMC8572062 34743243

[33] Mungur A Chen H Shahid S : A systematic review on the effect of silver diamine fluoride for management of dental caries in permanent teeth. *Clin. Exp. Dent. Res.* 2023;9:375–387. 10.1002/cre2.716 36823765 PMC10098297

[34] Bonifácio CC Hesse D : Is silver diammine fluoride effective in arresting dental caries in cavitated primary molars? *Evid. Based Dent.* 2023;24:50–51. 10.1038/s41432-023-00874-8 37130922

[35] Sharda S Gupta A Goyal A : Remineralization potential and caries preventive efficacy of CPP-ACP/Xylitol/Ozone/Bioactive glass and topical fluoride combined therapy versus fluoride mono-therapy–a systematic review and meta-analysis. *Acta Odontol. Scand.* 2021;79:402–417. 10.1080/00016357.2020.1869827 33459095

[36] Alkarad L Alkhouli M Dashash M : Remineralization of teeth with casein phosphopeptide-amorphous calcium phosphate: analysis of salivary pH and the rate of salivary flow. *BDJ open.* 2023;9:16. 10.1038/s41405-023-00141-z 37041136 PMC10090128

[37] Bakkal M Abbasoglu Z Kargul B : The Effect of Casein Phosphopeptide-Amorphous Calcium Phosphate on Molar-Incisor Hypomineralisation: A Pilot Study. *Oral Heal. Prev Dent.* 2017;15:163–167. 10.3290/j.ohpd.a37928 28322360

[38] Cardoso-Martins I Pessanha S Coelho A : Evaluation of the Efficacy of CPP-ACP Remineralizing Mousse in Molar-Incisor Hypomineralized Teeth Using Polarized Raman and Scanning Electron Microscopy—An in vitro Study. *Biomedicine.* 2022;10:3086. 10.3390/biomedicines10123086 PMC9775541 36551842

