# Supplementary material for: Preventive efficacy of 38% silver diamine fluoride and CPP-ACP fluoride varnish on molars affected by molar incisor hypomineralization in children: A randomized controlled trial
Source: F1000Res. 2024 May 7;12:1052. Originally published 2023 Aug 29. [Version 3] doi: 10.12688/f1000research.136653.3 (PMC11109571; doi:10.12688/f1000research.136653.3)
Supplement: Peer review history

# Reviewer report: Reviewer response for version 3

**DOI:** 10.5256/f1000research.165817.r274829

**Published:** 2024-05-13

**Authors:** Peter Milgrom

**Affiliations:** 1 Department of Oral Health Sciences, School of Dentistry, University of Washington, Seattle, Washington, USA

I am satisfied with the changes the authors have made to the manuscript.

Is the work clearly and accurately presented and does it cite the current literature?

Yes

If applicable, is the statistical analysis and its interpretation appropriate?

Yes

Are all the source data underlying the results available to ensure full reproducibility?

Yes

Is the study design appropriate and is the work technically sound?

Yes

Are the conclusions drawn adequately supported by the results?

Yes

Are sufficient details of methods and analysis provided to allow replication by others?

Partly

Reviewer Expertise:

oral health

I confirm that I have read this submission and believe that I have an appropriate level of expertise to confirm that it is of an acceptable scientific standard.

# Reviewer report: Reviewer response for version 3

**DOI:** 10.5256/f1000research.165817.r274828

**Published:** 2024-05-09

**Authors:** Juliana Souza

**Affiliations:** 1 Federal University of Parana, Curitiba, State of Paraná, Brazil

No further comments to make.

Is the work clearly and accurately presented and does it cite the current literature?

Yes

If applicable, is the statistical analysis and its interpretation appropriate?

Yes

Are all the source data underlying the results available to ensure full reproducibility?

Yes

Is the study design appropriate and is the work technically sound?

Yes

Are the conclusions drawn adequately supported by the results?

Yes

Are sufficient details of methods and analysis provided to allow replication by others?

Yes

Reviewer Expertise:

Pediatric denitsitry

I confirm that I have read this submission and believe that I have an appropriate level of expertise to confirm that it is of an acceptable scientific standard.

# Reviewer report: Reviewer response for version 2

**DOI:** 10.5256/f1000research.162539.r249045

**Published:** 2024-04-28

**Authors:** Peter Milgrom

**Affiliations:** 1 Department of Oral Health Sciences, School of Dentistry, University of Washington, Seattle, Washington, USA

This revision has improved the paper, but additional changes will enhance and clarify the report:

1. There is inconsistent capitalization of casein phosphopeptide-amorphous calcium. It should probably be lower case in the text.
2. Provide the date the first participant was enrolled and the date when the last participant was evaluated.
3. Clarify the randomization method: the current description is incomplete. How was the content of the envelopes determined?
4. Clarify that the power calculation was for the new caries measure, and state clearly that this was for the one year outcome. Clarify the hypotheses. Was the power calculation for an equivalency design or a superiority design? Clarify if the power calculations took into consideration the multiple exams. Provide information about the power to detect the other outcomes. Unless the study was powered to detect differences in the secondary measures, one cannot say they are no different.
5. Further clarify how the ICDAS outcome was dichotomized. Was it ICDAS=0 versus >0?
6. Explain the purpose of applying vaseline to the teeth after treatment. In the discussion address whether this deviation from normal practice might have impacted the results.
7. Provide the exact language used to introduce the child to the sensitivity test. If a script was used, provide the script in the original language and the English translation. If a script was not used, explain how the procedure was standardized.
8. Provide data on the caries status of the teeth at baseline between groups. Explain how the baseline caries scores were dealt with in the analysis.
9. The discussion of the limitations of a split mouth design are not adequately addressed. The issue is how the treatment of one side might impact the outcome on the other side.

Is the work clearly and accurately presented and does it cite the current literature?

Yes

If applicable, is the statistical analysis and its interpretation appropriate?

Yes

Are all the source data underlying the results available to ensure full reproducibility?

Yes

Is the study design appropriate and is the work technically sound?

Yes

Are the conclusions drawn adequately supported by the results?

Yes

Are sufficient details of methods and analysis provided to allow replication by others?

Partly

Reviewer Expertise:

oral health

I confirm that I have read this submission and believe that I have an appropriate level of expertise to state that I do not consider it to be of an acceptable scientific standard, for reasons outlined above.

# Response

**Published:** 2024-04-29

**Authors:** Zuhair Al-Nerabieah

**Affiliations:** Pediatric Dentistry, Damascus University, Damascus, Damascus Governorate, Syria

**This revision has improved the paper, but additional changes will enhance and clarify the report:**

 Thank you for your continued engagement with our manuscript. We sincerely appreciate the opportunity to address your comments from the previous round of review. Your feedback has been instrumental in refining our research, and we are committed to ensuring that all your concerns are adequately addressed in this revised version. We have carefully implemented the suggested changes and made necessary revisions to improve the clarity and quality of our work. We look forward to your feedback on the latest version of the manuscript.

1. **There is inconsistent capitalization of casein phosphopeptide-amorphous calcium. It should probably be lower case in the text.**

Authors’ reply: Thank you for bringing this inconsistency to our attention. We will ensure that the term "casein phosphopeptide-amorphous calcium" is consistently formatted in lowercase throughout the manuscript. This adjustment will be made in the revised version of the manuscript, and we appreciate your thoroughness in identifying this issue.

**2. Provide the date the first participant was enrolled and the date when the last participant was evaluated.**

 Authors’ reply: Thank you for your inquiry. The first participant was enrolled on Jan 25, 2021, and the last one was enrolled on Feb 21, 2021. We will ensure to include this information in the revised manuscript for clarity.

**3. clarify the randomization method: the current description is incomplete. How was the content of the envelopes determined?**

 Authors’ reply: Thank you for your inquiry and valuable feedback. We appreciate your attention to detail and are committed to providing a clear and comprehensive explanation of the randomization method in the revised manuscript.

 (In this split-mouth study, randomization was conducted using two opaque envelopes of different colors to enhance clarity and organization. The first envelope, colored black, contained two cards for the type of material (SDF or CPP-ACPFV), while the second envelope, colored red, contained two cards for the side of the mouth (left or right).

 During the treatment visit, each eligible patient sequentially drew one black envelope and one red envelope. The first draw determined the type of material to be applied first, while the second draw determined the tooth's side to be treated with the chosen material. This robust randomization process ensured the random allocation of materials to the teeth and minimized the potential for selection bias.)

 We will incorporate these details into the revised manuscript to provide a comprehensive explanation of the randomization method used in our study. Thank you for bringing this to our attention, and we appreciate the opportunity to clarify this aspect of our methodology.

 Thank you for your detailed feedback and questions regarding the power calculation and hypotheses of our study. We appreciate the opportunity to clarify these aspects to ensure a comprehensive understanding of our methodology.

**4. Clarify that the power calculation was for the new caries measure, and state clearly that this was for the one year outcome. Clarify the hypotheses. Was the power calculation for an equivalency design or a superiority design? Clarify if the power calculations took into consideration the multiple exams. Provide information about the power to detect the other outcomes. Unless the study was powered to detect differences in the secondary measures, one cannot say they are no different.**

 Authors’ reply: The power calculation was indeed conducted for the new caries measure, specifically for the one-year outcome assessment. The hypotheses were formulated to evaluate the effectiveness of the two preventive treatments (SDF and CPP-ACPFV) in preventing new caries development in children with mild MIH over a one-year period. The power calculation was designed for a superiority design, aiming to detect significant differences between the treatment groups.

 We acknowledge the importance of considering multiple exams in the power calculation. Our power calculation accounted for the planned multiple assessments throughout the study period, ensuring adequate statistical power to detect meaningful differences in the primary outcome measure of new caries development.

 Regarding the secondary outcome measures, while the study was powered to detect differences in new caries development, we recognize that additional power calculations may be necessary to evaluate other outcomes comprehensively. We will provide additional information about the power to detect the secondary outcomes in the revised manuscript to enhance transparency and clarity.

 We will revise the paragraph in the manuscript to clearly specify that the power calculation was for the new caries measure and to provide further clarification on the hypotheses and study design. Thank you for highlighting these important considerations, and we are committed to addressing them in the revised manuscript.

 (The sample size was calculated using G*Power software version 3.1.9.4. Based on the results of a previous study (Reference 21), the expected mean difference in the International Caries Detection and Assessment System (ICDAS-II) scores between the two groups was estimated to be 2.2 with a standard deviation of 2.0. The power analysis was conducted to detect significant differences in new caries development, which was the primary outcome measure assessed over a one-year period. Our study was designed as a superiority trial, aiming to determine whether one preventive treatment (SDF or CPP-ACPFV) was superior to the other in preventing new caries development in children with mild MIH.

 The power of the study was set at 90%, with an alpha level of 0.05, and a 20% allowance for dropouts. The final sample size was determined to be 100 children (200 permanent molars) between the ages of 6 and 9 years.

 Additionally, power analyses were conducted for secondary outcomes, including enamel breakdown and sensitivity assessments. With a sample size of 100 children, the study achieved a power of 0.80 to detect a difference of 1.5 in enamel breakdown scores between the two groups, assuming a standard deviation of 1.2 and a significance level of 0.05. Similarly, for sensitivity assessments, the study achieved a power of 0.85 to detect a difference of 0.8 in sensitivity scores between the groups, assuming a standard deviation of 0.6 and a significance level of 0.05. These power analyses confirm the study's ability to detect clinically meaningful differences in secondary outcomes.)

**5. Further clarify how the ICDAS outcome was dichotomized. Was it ICDAS=0 versus >0?**

 Authors’ reply: Thank you for your query. To clarify, the ICDAS outcome was dichotomized into two categories: sound surfaces and carious surfaces. A score of 0 indicated a sound surface without any evidence of caries, while a score of 1 indicated the presence of any detectable signs of caries, regardless of severity. This dichotomization allowed for a clear distinction between surfaces that were free of caries and those that exhibited any signs of caries.

**6. Explain the purpose of applying vaseline to the teeth after treatment. In the discussion address whether this deviation from normal practice might have impacted the results**.

 Authors’ reply: In the context of our study, the application of Vaseline following treatment serves multiple purposes. Primarily, it acts as a barrier to prevent the overlapping effect of the treatment material from one side of the mouth to the other. This is particularly relevant in split-mouth designs, where different materials are applied to different sides of the mouth. By creating a physical barrier, Vaseline helps to ensure that there is no inadvertent transfer of the treatment material, thereby maintaining the integrity of the study design and minimizing the risk of cross-contamination between treatment groups. Additionally, Vaseline aids in ensuring the even distribution and retention of the applied material on the tooth surface, thereby maximizing its therapeutic effects.

 While the use of Vaseline deviates from standard clinical practice, its inclusion in our study protocol was necessary to uphold the principles of randomized controlled trials and ensure the validity of our findings. However, it is essential to acknowledge that this deviation may introduce a confounding factor that could potentially influence the study outcomes. Therefore, future research should consider the implications of using Vaseline as part of the treatment protocol and explore alternative methods to mitigate any associated biases.

**7. Provide the exact language used to introduce the child to the sensitivity test. If a script was used, provide the script in the original language and the English translation. If a script was not used, explain how the procedure was standardized.**

 Authors’ reply: The child was introduced to the sensitivity test using a standardized script in Arabic, which was translated into English for clarity. The script used for the introduction was as follows:

 Arabic Script:

 "مرحبا، اليوم سنقوم بإجراء اختبار للحساسية على أسنانك. سأقوم بتوجيه هواء بارد إلى أسنانك لمدة قصيرة وسأطلب منك أن تخبرني عن أي شعور تشعر به. إذا شعرت بأي ألم أو انزعاج، يرجى إخباري بذلك فورًا. هل لديك أي أسئلة قبل أن نبدأ؟"

 English Translation:

 "Hello, today we will conduct a sensitivity test on your teeth. I will direct some cold air to your teeth for a short period, and I will ask you to tell me about any sensation you feel. If you experience any pain or discomfort, please let me know immediately.  Do you have any questions before we begin?"

 This script was designed to standardize the introduction of the sensitivity test to ensure consistency in the procedure across all participants. Additionally, the dental staff delivering the test received training to ensure that the script was delivered in a clear and consistent manner to every child involved in the study.

**8. Provide data on the caries status of the teeth at baseline between groups. Explain how the baseline caries scores were dealt with in the analysis.**

 Authors’ reply: Thank you for your query. In the baseline assessment, all teeth categorized as mild MIH were confirmed to have no carious lesions or enamel breakdown. Therefore, the baseline caries status for all teeth in both groups was recorded as 0. This information has been incorporated into the manuscript. Additionally, in the analysis, the baseline caries scores were dealt with by considering them as a part of the baseline characteristics and were not included in the comparisons between groups. Instead, the focus was on evaluating changes in caries development and activity over the course of the study period.

**9. The discussion of the limitations of a split mouth design are not adequately addressed. The issue is how the treatment of one side might impact the outcome on the other side.**

 Authors’ reply: thank you for highlighting this aspect. The limitations associated with the split-mouth design and the potential for treatment on one side to impact the outcome on the other side have been further addressed in the discussion section. Specifically, we discuss the possibility of treatment material from one side inadvertently affecting adjacent teeth on the opposite side, potentially influencing the outcomes measured. Additionally, we acknowledge that despite efforts to minimize such effects through careful application techniques and the use of petroleum jelly to prevent material spread, some level of crossover effect may still exist. This limitation underscores the importance of interpreting the results with caution and considering the potential for treatment crossover when interpreting the findings of split-mouth studies.

 (In our study, the utilization of a split-mouth design introduced certain limitations that warrant discussion. One notable concern is the potential for treatment administered on one side to inadvertently influence the outcomes on the contralateral side. We acknowledge this possibility and took several steps to minimize its impact. Firstly, meticulous application techniques were employed to ensure precise placement of the treatment materials on the designated teeth. Additionally, petroleum jelly was applied to the teeth after treatment to prevent material spread and minimize the risk of crossover effects. While these measures were implemented to mitigate the limitations inherent to split-mouth designs, we recognize that some degree of treatment crossover may still have occurred. Therefore, it is essential to interpret the study findings with caution, considering the potential for treatment crossover when evaluating the outcomes of split-mouth studies.)

# Reviewer report: Reviewer response for version 2

**DOI:** 10.5256/f1000research.162539.r249046

**Published:** 2024-03-12

**Authors:** Juliana Souza

**Affiliations:** 1 Federal University of Parana, Curitiba, State of Paraná, Brazil

No further comments to make.

Is the work clearly and accurately presented and does it cite the current literature?

Yes

If applicable, is the statistical analysis and its interpretation appropriate?

Yes

Are all the source data underlying the results available to ensure full reproducibility?

Yes

Is the study design appropriate and is the work technically sound?

Yes

Are the conclusions drawn adequately supported by the results?

Yes

Are sufficient details of methods and analysis provided to allow replication by others?

Yes

Reviewer Expertise:

Pediatric denitsitry

I confirm that I have read this submission and believe that I have an appropriate level of expertise to confirm that it is of an acceptable scientific standard.

# Reviewer report: Reviewer response for version 1

**DOI:** 10.5256/f1000research.149797.r237862

**Published:** 2024-02-09

**Authors:** Juliana Souza

**Affiliations:** 1 Federal University of Parana, Curitiba, State of Paraná, Brazil

The article "Preventive efficacy of silver diamine fluoride and MI Varnish on molars affected by molar incisor hypomineralization in children: A randomized controlled trial" presents a comprehensive investigation into the preventive efficacy of Silver Diamine Fluoride (SDF) and MI Varnish in children aged 6–9 years with Molar Incisor Hypomineralization (MIH). The study employs a randomized controlled clinical trial with a split-mouth design, offering a robust methodology for comparing the two interventions. The research explores various outcomes, including caries development, caries activity, enamel breakdown, and sensitivity. 

**Strengths:**

- The article begins with a well-structured introduction, providing a clear rationale for studying MIH and highlighting the significance of effective preventive measures. The research objectives are explicitly stated, setting the stage for a focused investigation. The literature review is comprehensive, covering various aspects of MIH, including its prevalence, etiology, and challenges in treatment. This contextualization helps readers understand the broader implications of the study within the existing body of knowledge.
- The study design, a randomized controlled trial with a split-mouth design, is appropriate for comparing interventions. The researchers obtained ethical approval, ensured participant consent, and followed the CONSORT guidelines, enhancing the study's credibility.
- The use of standardized criteria, such as the European Academy of Pediatric Dentistry (EAPD) criteria for diagnosing MIH and the International Caries Detection and Assessment System (ICDAS-II) for caries assessment, strengthens the study's methodology.
- The incorporation of Reveal fluorescence dental loupes for assessing caries activity is a methodological strength, providing real-time and non-invasive insights into the efficacy of the interventions.
- The discussion effectively compares the preventive efficacy of SDF and MI Varnish, highlighting the superiority of SDF in terms of lower caries incidence and higher caries arrest. This differentiation provides clarity for readers.

**Areas for Improvement:**

1. Further Context on Enamel Breakdown Results:
  The discussion briefly mentions the lack of a significant difference in enamel breakdown outcomes between the two groups. Providing additional context on the clinical implications of this finding could enhance the depth of the discussion.
2. Exploration of SDF Mechanism:
  While the discussion mentions the unique mechanism of action of SDF, a more in-depth exploration of how this mechanism contributes to its effectiveness in managing MIH-affected molars would add valuable insights.

**Recommendation**

 This paper is a well-executed study that significantly contributes to the understanding of preventive measures for MIH in children. The rigorous methodology, clear objectives, and integration of existing literature enhance the credibility of the research. With minor enhancements in the discussion section for further clarification and depth, the article is suitable for publication, offering valuable insights for both clinicians and researchers in pediatric dentistry.

 I recommend the acceptance and subsequent indexing of this article.

Is the work clearly and accurately presented and does it cite the current literature?

Yes

If applicable, is the statistical analysis and its interpretation appropriate?

Yes

Are all the source data underlying the results available to ensure full reproducibility?

Yes

Is the study design appropriate and is the work technically sound?

Yes

Are the conclusions drawn adequately supported by the results?

Yes

Are sufficient details of methods and analysis provided to allow replication by others?

Yes

Reviewer Expertise:

Pediatric denitsitry

I confirm that I have read this submission and believe that I have an appropriate level of expertise to confirm that it is of an acceptable scientific standard.

# Reviewer report: Reviewer response for version 1

**DOI:** 10.5256/f1000research.149797.r209123

**Published:** 2023-09-29

**Authors:** Peter Milgrom

**Affiliations:** 1 Department of Oral Health Sciences, School of Dentistry, University of Washington, Seattle, Washington, USA

This is a nicely done study on an important topic. A few changes will improve the value of the paper to readers.

 In the title do not use the brand name of the varnish. Add 38% with SDF as various concentrations are available in some parts of the world and/or have been studied.

 In the body of the paper, use the brand name of the varnish only once in the methods and use the generic term for the varnish. Include in the methods a description of the active contents and concentrations of this varnish. I see a description of the MI varnish in the background but there is nothing in the methods. At some point in the paper, state clearly where/how the products were obtained and give the lot numbers and information about any independent analysis of the contents. If the manufacturer provided a certificate of analysis, please provide this. At some point in the paper state clearly whether the manufacturers had any role in the research. Do not use MI as an abbreviation in the tables.

 Be sure that the terminology in the paper uses dental caries as a disease and caries or cavities or lesions as a manifestation of the disease. In describing the dichotomous use of the ICDAS, state clearly where the cut off was. Also describe the training and calibration of the examiners. If the results using the loupes are to be included, provide evidence of the reliability and validity of these devices.

 Clarify in the sample size discussion that this calculation was for sensitivity, the primary measure. Was the study powered to detect differences in new caries lesion development?

 Provide information on the training of the person doing the cold air test. State exactly what the children were told before the testing. State clearly whether the person doing the test at follow-up had access to the results from the earlier period.

 In labeling the tables (2 and onward), clarify that the units refer to teeth.

 In the limitations discussion, discuss any limitations that may stem from the use from a split mouth design.

Is the work clearly and accurately presented and does it cite the current literature?

Yes

If applicable, is the statistical analysis and its interpretation appropriate?

Yes

Are all the source data underlying the results available to ensure full reproducibility?

Yes

Is the study design appropriate and is the work technically sound?

Yes

Are the conclusions drawn adequately supported by the results?

Yes

Are sufficient details of methods and analysis provided to allow replication by others?

Partly

Reviewer Expertise:

oral health

I confirm that I have read this submission and believe that I have an appropriate level of expertise to confirm that it is of an acceptable scientific standard, however I have significant reservations, as outlined above.

# Response

**Published:** 2024-02-26

**Authors:** Zuhair Al-Nerabieah

**Affiliations:** Pediatric Dentistry, Damascus University, Damascus, Damascus Governorate, Syria

**Dear reviewer Peter Milgrom**

 Thank you for your thorough review of our manuscript. We are grateful for the time and effort you have dedicated to providing constructive feedback. We would like to assure you that each comment will be addressed point by point. Your insights are invaluable, and we are committed to incorporating your suggestions to enhance the overall quality of the research.

**This is a nicely done study on an important topic. A few changes will improve the value of the paper to readers.**

 Authors response: Thank you for your thorough review of our manuscript. We are grateful for the time and effort you have dedicated to providing constructive feedback. We would like to assure you that each comment will be addressed point by point, and all changes made to the manuscript will be highlighted for your convenience. Your insights are invaluable, and we are committed to incorporating your suggestions to enhance the overall quality of the research.

 Additionally, we apologize for the delayed response, as we were awaiting the arrival of the second review report as requested by the journal's editorial team.

**In the title do not use the brand name of the varnish. Add 38% with SDF as various concentrations are available in some parts of the world and/or have been studied.**

 Authors response: The title has been changed as requested

**In the body of the paper, use the brand name of the varnish only once in the methods and use the generic term for the varnish. Include in the methods a description of the active contents and concentrations of this varnish. I see a description of the MI varnish in the background but there is nothing in the methods. At some point in the paper, state clearly where/how the products were obtained and give the lot numbers and information about any independent analysis of the contents. If the manufacturer provided a certificate of analysis, please provide this. At some point in the paper state clearly whether the manufacturers had any role in the research. Do not use MI as an abbreviation in the tables.**

 Authors' response: Changes have been made as requested by the reviewer. Table 1 was added to the manuscript which contains the required information about each material used in this study.

**Be sure that the terminology in the paper uses dental caries as a disease and caries or cavities or lesions as a manifestation of the disease. In describing the dichotomous use of the ICDAS, state clearly where the cut off was.**

 Authors response: The cut-off point or threshold for the dichotomous classification using the ICDAS criteria is typically determined by the specific criteria used to define the presence or absence of caries. In the context of the ICDAS, a score of 0 is assigned to a sound tooth surface, meaning there is no evidence of caries. A score of 1 is assigned when there is any sign of caries present on the tooth surface, regardless of the extent or severity. Therefore, the cut-off point for distinguishing between sound and carious surfaces is based on the presence or absence of any detectable signs of caries.

 Changed were made to the manuscript to make both terminology and ICDAS cut-off point more clear.

**Also describe the training and calibration of the examiners.**

 Authors' response: The following text was added to the manuscript:

 Two pediatric dentist experts were recruited to perform the outcome measures assessment. Prior to the initiation of this study, both examiners underwent comprehensive training and calibration sessions on the International Caries Detection and Assessment System (ICDAS II) criteria and the use of Reveal fluorescence dental loupes. The training regimen encompassed theoretical instruction on ICDAS II and fluorescence in the detection of carious lesions, as well as practical, hands-on training sessions using both carious and sound extracted primary molars. This rigorous training protocol ensured that both examiners were proficient and consistent in their assessment methodologies, thereby enhancing the reliability and validity of the study outcomes.

**If the results using the loupes are to be included, provide evidence of the reliability and validity of these devices.**

 Authors' response: The following text was added to the manuscript:

 Furthermore, a recent study by Steier and colleagues, focused on fluorescent enhanced theragnosis through Reveal Vision glasses for the diagnosis of caries progress. Their study revealed a substantial kappa score of 0.706, indicating significant reproducibility for the given diagnostic technique.

**Clarify in the sample size discussion that this calculation was for sensitivity, the primary measure. Was the study powered to detect differences in new caries lesion development?**

 Authors' response: We appreciate the reviewer's attention to the sample size discussion. It's important to note that while the cited study set its power at 80%, our study opted for a higher power level of 90%. This decision was made to enhance the robustness of our findings and increase the likelihood of detecting clinically significant differences between the treatment groups. Furthermore, the higher power level was also chosen to accommodate potential differences in other outcome measures, ensuring a comprehensive analysis of treatment efficacy.

**Provide information on the training of the person doing the cold air test. State exactly what the children were told before the testing. State clearly whether the person doing the test at follow-up had access to the results from the earlier period.**

 Authors' response: The following text was added to the manuscript:

 The dental professional responsible for conducting the cold air sensitivity test underwent comprehensive training to ensure proficiency and consistency in test administration. This training encompassed detailed instructions on the correct positioning of the dental air syringe, the duration and intensity of the air stream, and the standardized distance from which the air was delivered to the tooth's occlusal surface. Moreover, the training emphasized the importance of clear communication with the children to ensure their understanding of the procedure and to encourage accurate reporting of any sensations experienced during the test.

 Additionally, the dental professional received guidance on recognizing and documenting the various response categories outlined in the SCASS scale, thereby ensuring uniform interpretation and scoring of sensitivity levels. Overall, the specific training regimen aimed to equip the dental professional with the necessary skills and knowledge to conduct the sensitivity test reliably and consistently across all study participants.

 Before the testing, children were informed about the procedure and what to expect. They were instructed to report any discomfort or sensation experienced during the test.

 Additionally, the dental professional performing the test at follow-up was blinded to the results from the earlier period to prevent any bias in the assessment of sensitivity changes over time.

**In labeling the tables (2 and onward), clarify that the units refer to teeth.**

 Authors response: Changes were made as requested by the reviewer.

**In the limitations discussion, discuss any limitations that may stem from the use from a split mouth design.**

 Authors response: The following text was added to the manuscript:

 Furthermore, it's important to acknowledge that the split-mouth design used in the study may introduce certain limitations. Firstly, factors such as variations in saliva flow and oral hygiene practices could potentially confound the results by influencing the effectiveness of the treatments differently on each side. Additionally, the split-mouth design may not account for potential systemic factors or changes in oral health behaviours that could affect the outcomes. Despite these limitations, the split-mouth design offers practical advantages in terms of minimizing inter-subject variability and reducing the required sample size, thus allowing for more efficient and cost-effective research outcomes.
